# Organ motion in pediatric high‐risk neuroblastoma patients using four‐dimensional computed tomography

**DOI:** 10.1002/acm2.12012

**Published:** 2016-12-10

**Authors:** Sneha Kannan, Boon‐Keng Kevin Teo, Timothy Solberg, Christine Hill‐Kayser

**Affiliations:** ^1^ Department of Radiation Oncology University of Pennsylvania Philadelphia PA 19104 USA

**Keywords:** 4DCT, neuroblastoma, organ motion, pediatric

## Abstract

**Purpose/objective(s):**

High‐risk neuroblastoma (HR‐NBL) requires multimodality treatment, including external beam radiation of the primary tumor site following resection. Radiotherapy planning must take into account motion of the target and adjacent normal anatomy, both of which are poorly understood in the pediatric population, and which may differ significantly from those in adults.

**Methods/materials:**

We examined 4DCT scans of 15 consecutive pediatric patients treated for HR‐NBL, most with tumors in the abdominal cavity. The diaphragm and organs at risk were contoured at full inhale, full exhale, and on free‐breathing scans. Maximum displacement of organs between full inhale and full exhale was measured in the anterior, posterior, superior, inferior, left, and right directions, as was displacement of centroids in the A/P, S/I, and L/R axes. Contours on free‐breathing scans were compared to those on 4D scans.

**Results:**

Maximum displacement was along the S/I axis, with the superior aspects of organs moving more than the inferior, implying organ compression with respiration. Liver and spleen exhibited the largest motion, which correlated strongly with the S/I motion of the diaphragm. The maximum organ motion observed in the abdomen and thorax were 4.5 mm and 7.4 mm, respectively, while maximum diaphragm displacement was 5.7 mm. Overall findings mirrored observations in adults, but with smaller magnitudes, as expected. No consistent margins could be added to the free‐breathing scans to encompass the motion determined using 4DCT.

**Conclusions:**

Organ motion within the pediatric abdomen and pelvis is similar to that observed in adults, but with smaller magnitude. Precise margins to accommodate motion are patient‐specific, underscoring the need for 4DCT scanning when possible.

## 1 Introduction

Modern treatment paradigms for HR‐NBL include external beam radiotherapy (EBRT) to the primary tumor site and refractory metastatic sites in combination with intense multimodality therapy. Primary neuroblastoma tumors can occur along various points in the peripheral nervous system, and necessary radiation treatment volumes may be complex and in very close proximity to lungs, heart, liver, kidneys, and diaphragm. As a result, many organs may be at risk or compromised by the presence of tumor, surgical necessity, or subsequent radiation. Additionally, secondary cancers have been shown to arise in regions of healthy tissue exposed to radiation.^1^ For all of these reasons, striking an optimal balance between radiating areas at risk for microscopic disease, yet minimizing normal tissue exposure, is of paramount importance.

In recent years, more complex forms of EBRT, including intensity modulated x‐ray therapy (IMRT) and proton therapy (PT), have become important in treatment of HR‐NBL.^2^, ^3^, ^4^ Unlike therapeutic x‐rays, the physical properties of PT allow proton beams to stop within a predetermined margin of the target, maximizing preservation of normal tissue. Proton therapy has been shown to reduce radiation dose to healthy organs while maintaining target coverage in HR‐NBL children.^2^, ^3^ As treatments become more precise, however, understanding organ motion within this young population becomes increasingly important. Liver, lung, and kidney are recognized to move with every breathing cycle, and quantifying this movement is essential during EBRT planning.^5^

4DCT represents a unique technology to assess organ motion from respiration and other normal physiology. Although 4DCT has been used extensively to examine organ motion in adults, similar analysis in the pediatric population is lacking. This is because there are few clinical indications for using 4DCT in this population, but as more precise treatment modalities (e.g., proton therapy) are developed, treatment techniques that may benefit from 4DCT, which provides more information about organ motion and physiology, may become more common. Furthermore, because 4DCT scans involves a higher imaging dose, there may be reluctance to apply the technique in the pediatric population. Further information about our institution's protocol is included in the Methods section below. Ultimately the desire to avoid additional imaging dose in the pediatric population should be balanced with better treatment planning and delivery, which could better spare normal structures.

During EBRT planning, gross tumor volume (GTV) and clinical target volume (CTV) are delineated, and physiologic motion of tumor and organs at risk (OAR) are incorporated along with variation in patient position (the planning target volume — PTV) on a daily basis. If 4DCT is available, treatment margins may be generated from information determined from the inhale and exhale scans (which, as this work shows, encompass all motion); however, many centers either do not have access to 4DCT or do not perform 4DCT in the pediatric population, and thus may use standard margins derived from adult patients, which vary with institution. No consensus target or normal organ margins that take normal physiologic motion into account exist for the pediatric population, and those used for adult patients may be larger than needed for very small children. This approach is expected to result in unnecessary extra exposure of normal OARs. In contrast, expansions that are not large enough to adequately take motion into account could result in a region involved with tumor being outside the high‐dose radiation volume. Finally, accurate estimation of organ position within various phases of the respiratory cycle allows improved understanding of normal organ radiation exposure. The rationale for this study was to utilize 4DCT to analyze normal organ motion and its relationship with the breathing cycle, and to model necessary expansions for OARs in a population of pediatric neuroblastoma patients.

## 2 Methods

Data from 15 pediatric patients with high‐risk neuroblastoma who received radiotherapy between June 2011 and December 2014 were analyzed. Ages ranged from 1.5–10 years. All had received systemic treatment surgical resection according to the current treatment paradigm. All received EBRT to the primary tumor site‐1 in the thoracic cavity and 14 in the abdominal cavity. All underwent CT simulation with 4DCT as part of routine clinical care using a low‐dose protocol for proton treatment. No additional scans were acquired as part of this study. These patients received radiation post‐operatively following resection of the primary tumor. As a result, the scans used for treatment planning did not contain the initial tumor. Thus, tumor volumes were contoured based on preoperative images that were fused with the 4D planning CT images.

At our institution, the tube current is reduced and tube current modulation is used to reduce dose for all 4DCT scans. The resulting CT dose index (CTDI) for 4DCT scans is approximately 1.6 times that of a conventional CT scan. While individual phases of the 4DCT scan are of inferior quality compared to a conventional scan, the image quality is sufficient for contouring purposes and to assess motion. The treatment plan is calculated on the average of the phases, which is of comparable quality to a regular CT.

Organs at risk were contoured (Table 1). For patients with abdominal tumors, these generally included kidneys, liver, spleen, and diaphragms. For patients with thoracic tumors (or occasionally adrenal tumors when scans were limited inferiorly), they included lungs, heart, and diaphragms. One patient had a kidney removed previously, and some patients also had 4D imaging that did not encompass all organs of interest. 4DCT images were acquired on a Siemens Sensation Open scanner (Siemens Medical Solutions, Malvern, PA, USA) using a low‐pitch (0.1) helical mode. The slice thickness was 3 mm in the 4D scans and 1.5 mm in the free‐breathing scans. Respiratory traces were obtained using the Realtime Position Management (RPM) system (Varian Medical Systems, Palo Alto, CA, USA). The RPM utilizes a camera system to capture the vertical displacement of a marker block placed on the patients' sternum. CT data were binned into eight uniform phases. Maximum inhalation was defined as the 0% phase and maximum exhalation was typically close to the 50% phase. Patients were positioned supine using a Vac‐Lok bag for immobilization; an Aquaplast mask was added for patients treated for thoracic tumors. Ten patients were simulated and treated under general anesthesia; the five did not require anesthesia and did not receive coaching for breath regularity. All contours were created within Eclipse (version 11.0; Varian Medical Systems, Palo Alto, CA, USA) and position measurements were measured using the software's measuring tool.

**Table 1:**
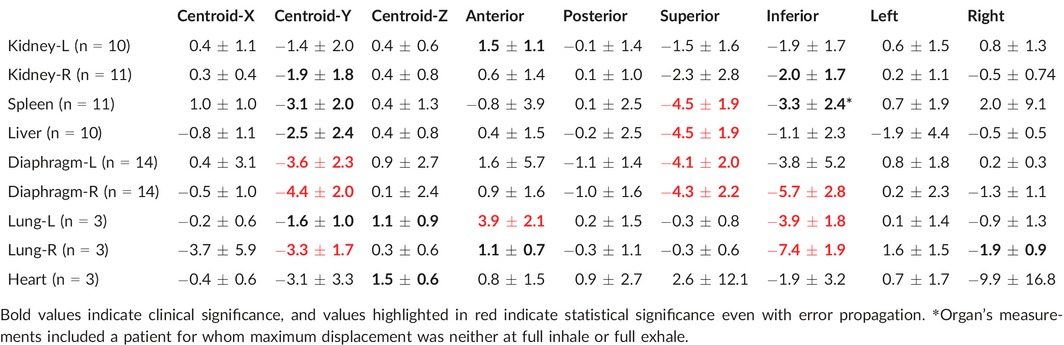
Mean ± SD organ (in mm) displacement between maximum inhale and maximum exhale across extremes of three major axes. Positive displacement indicates INHALE was more extreme than EXHALE. Centroid‐X represents movement of the centroid in the L/R direction, Y in the S/I direction, Z in the A/P direction

To minimize variability, all organs were contoured by a single investigator. As the diaphragm is continuous with the inferior aspect of the lung, diaphragms were contoured by extending the lung contours inferiorly by 3 mm,^6^, ^7^ and the lung volume was subsequently subtracted. The contours were manually smoothed and then postprocessed to remove all contours with an area< 0.5 cm^2^. Right and left diaphragms/lungs were contoured and evaluated separately. Kidneys were contoured without separating the cortex and medulla, and excluded the hilum. The heart was contoured beginning superiorly with the appearance of the right ventricle, and including the pericardium and all of its contents.

Organs were contoured on maximum inspiration and expiration phases, as well as on the free‐breathing scans. The most extreme inferior, superior, anterior, posterior, left and right points were evaluated by measuring the maximum edge of each of the three main axes (Left/Right‐X, Superior/Inferior‐Y, Anterior/Posterior‐Z) in each organ. The resolution of the measuring tool (0.6 mm) was smaller than the slice thickness (1.5 mm or 3.0 mm), so measurements in the superior/inferior direction were performed by manually acquiring the coordinates for the points of interest.

Given a measurement resolution of 0.6 mm in the contouring software, error was accordingly propagated to accommodate the difference between two edges (i.e., displacement) (mean ± 0.6 mm and SD + 0.8 mm based on the propagating variance for two variables). The current standard in literature is to define clinically relevant organ motion as that where the mean for a population exceeds its standard deviation (i.e., 1.0 ± 0.5 cm), which has been taken to broadly mean that there is a trend for an organ to move in specific direction. Though error propagation has been uncommon in previous work, we chose to include it in order to allow for quantification of the uncertainty introduced by contouring, and determination of whether the criteria of SD < Mean was met after accounting for measurement imprecision. Cells with red text in Tables 1 and 2 show the measurements that showed a trend after error propagation, while bolded values indicate measurements that were clinically relevant according to current standards in literature.

**Table 2:**
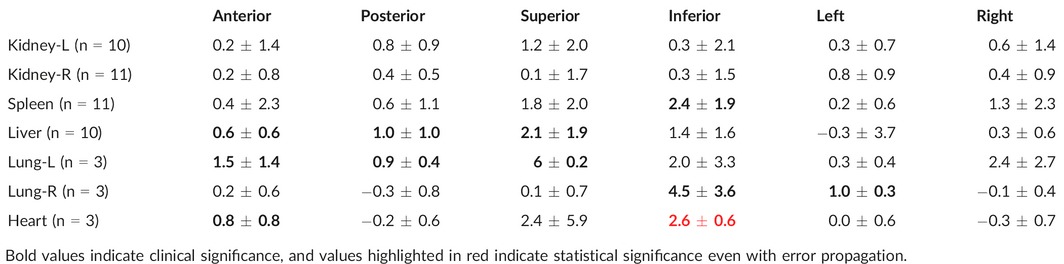
Mean ± SD margins (in mm) added to free‐breathing contours in order to contain organs in all 4D breathing phases

Previous work has indicated that the full inhale and full exhale tend to encompass the most extremes of motion,^8^, ^9^ and contours encompassing both maximum inhale and exhale were created (i.e., the union of organ structures). Conversely, the use of maximum intensity projection (MIP) images can induce inaccuracies in motion assessment, particularly for irregular breathing patterns.^10^, ^11^ Organs in every other breathing phase were assessed to ensure that the union structure fully encompassed each organ's extreme positions in all breathing phases.

**Figure 1:**
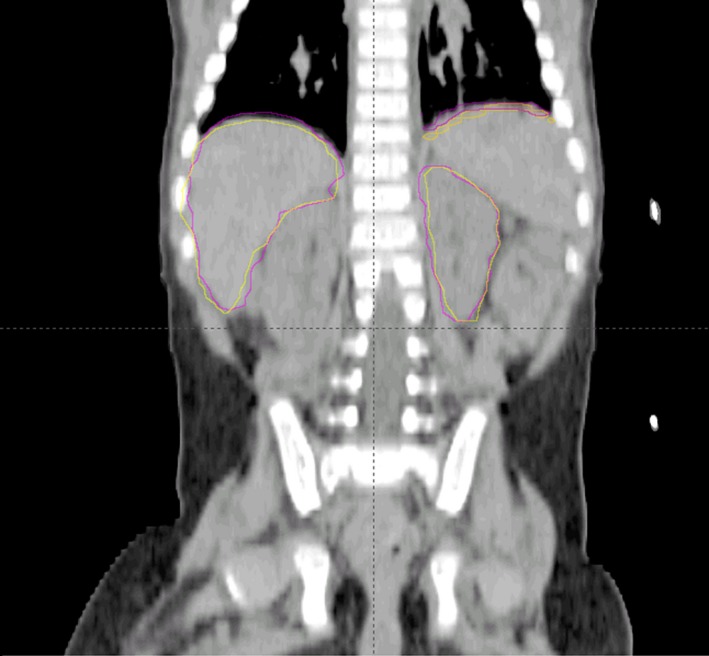
Schematic of margin analysis done on free‐breathing and 4DCT contours. The center is the free‐breathing organ. Free‐breathing organ contours were analyzed to see if reliable margins, as shown in the figure, could be added to accommodate the union of maximum inhale and maximum exhale (the most extreme positions of organs in 4DCT).

Patients in this series underwent both 4DCT and free‐breathing scans. The 4DCT scans focused on the diaphragm and the target area (either abdomen or thorax). On free‐breathing scans, lungs, heart, kidneys, liver, and spleen were contoured, and the most extreme LR, AP, and SI positions determined. These values were compared to inhale and exhale positions on 4DCT to determine the length that would have to be added in each of the six directions to fully encompass the organ's most extreme position in each of six directions during all 4DCT breathing phases (Fig. 1). This was measured by determining the difference between the organ during the free‐breathing scan and the maximum of inhale or exhale in any given direction.

Patient free‐breathing CT organ motion data and inhale/exhale differences were analyzed individually, and in groups based on age, abdominal surgical history, and anesthesia status.

## 3 Results

**Figure 2:**
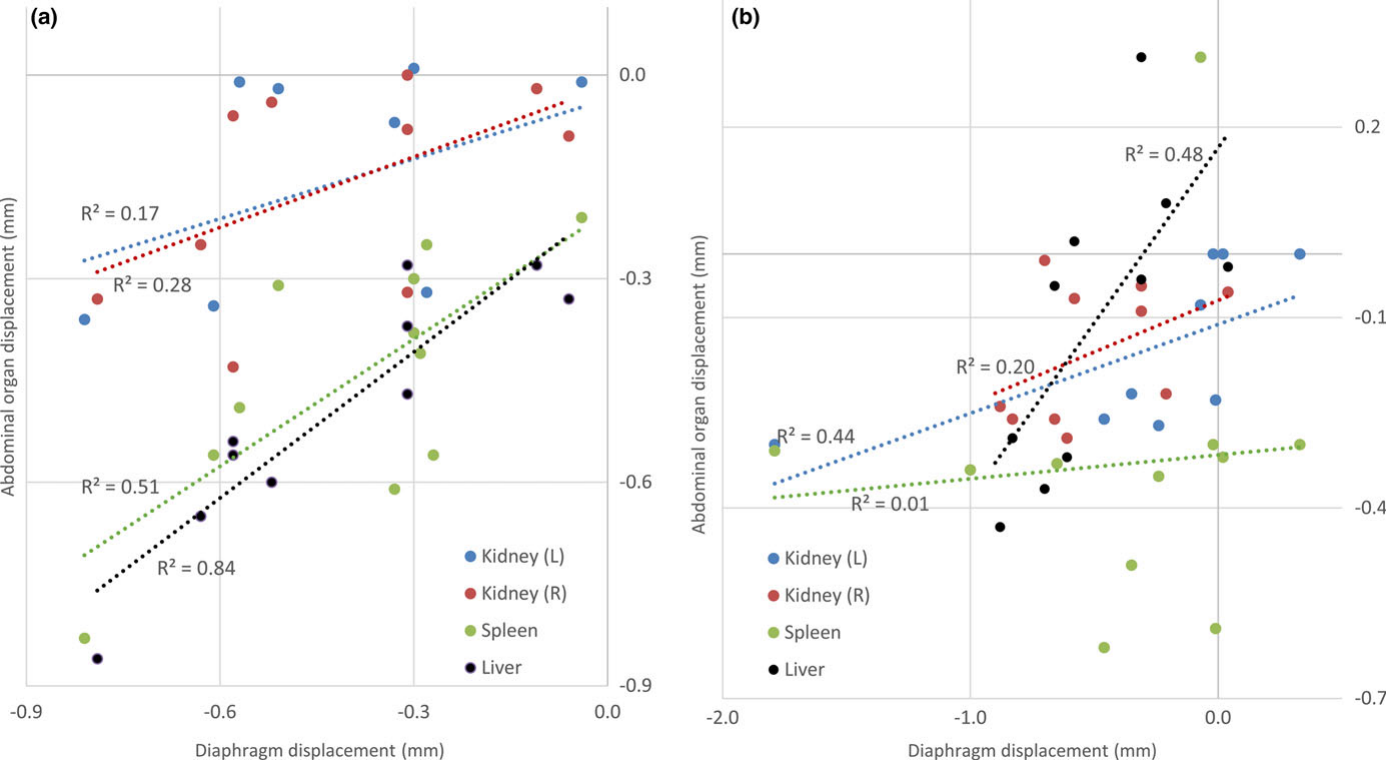
Coronal slice with L diaphragm, L kidney, and liver contours on inhale (yellow) and exhale (purple).

In total, organ motion was evaluated in 15 patients (average age 4.2 years); 14 had a primary tumor in the abdominal cavity and 1 in the thoracic cavity. Figure 2 shows a sample coronal slice with left diaphragm inhale and exhale with an example of a kidney and liver contour on one patient.

All organs were analyzed for the maximum displacement in all directions. The most extreme AP, LR, and SI positions between inhale and exhale were then compared (Table 1). The centroid position and volume in cm^3^ were also recorded, with differences shown in (Table 1). With the exception of the kidneys, the superior aspect of organs uniformly moved inferiorly during inhale, as expected. Abdominal organs moved together, with each showing superior displacement similar in magnitude. Abdominal organ inferior edge motion was less consistent as was centroid motion in the Y direction. Overall, most organs showed greater displacement of the superior edge as compared to the inferior edge. The lungs showed moderate anterior motion, which was reflected to a lesser degree in centroid‐Z motion.

**Figure 3:**
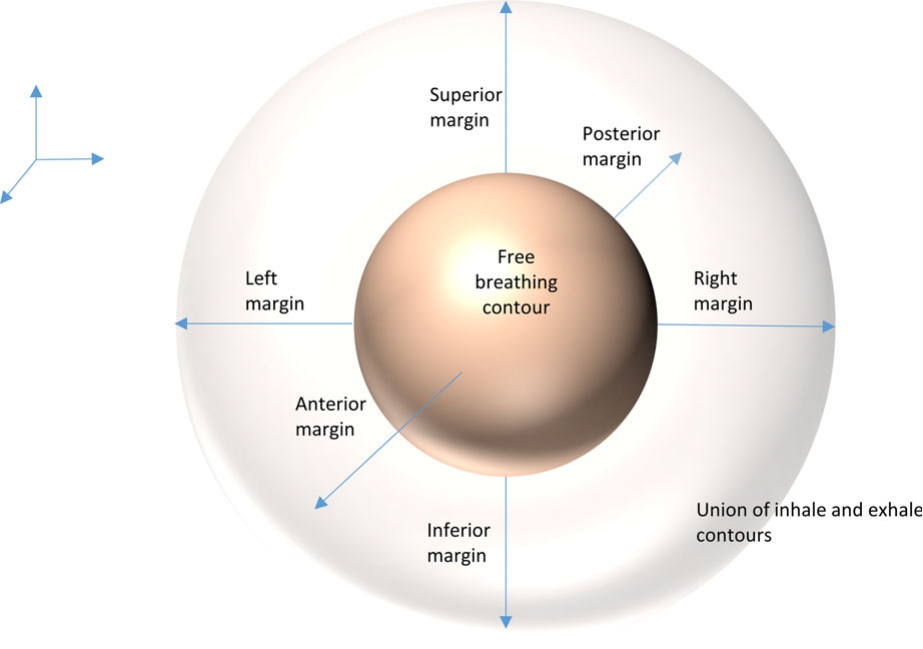
Displacement of abdominal organs versus diaphragm displacement in: (a) superior and (b) inferior directions.

Table 2 shows a comparison of the free‐breathing organ contours to the 4DCT contours. With the exception of one patient whose spleen reached maximum inferior position at the 87% breathing phase (Table 1, see asterisk), the union of the inhale and exhale contours encompassed the organ position extremes at all breathing phases and on all axes. It is notable that no consistent and precise margin could be added to the free‐breathing scans to encompass motion observed on the 4D scans, especially after error propagation; however, at the positional extremes, abdominal organs differed no more than 6.3 mm between free‐breathing and 4D scans and thoracic organs no more than 9.2 mm. Figure 3 shows the INH‐EXH displacements of the abdominal organs versus the corresponding diaphragms. Linear regressions and corresponding R^2^ values are also included. In the superior direction, which showed more consistent and larger displacements, the correlations are strong for liver (R^2^ = 0.84) and spleen (R^2^ = 0.51). The kidneys showed weaker R^2^ values (0.17 for the left and 0.28 for the right). In the inferior direction, the correlations are less strong. The liver, again, showed a strong correlation with an R^2^ = 0.48. The spleen showed almost no correlation between diaphragm motion and organ motion in the inferior direction. The right kidney showed a similar correlation while the left kidney showed a much stronger correlation in the inferior direction compared to the superior direction.

**Figure 4:**
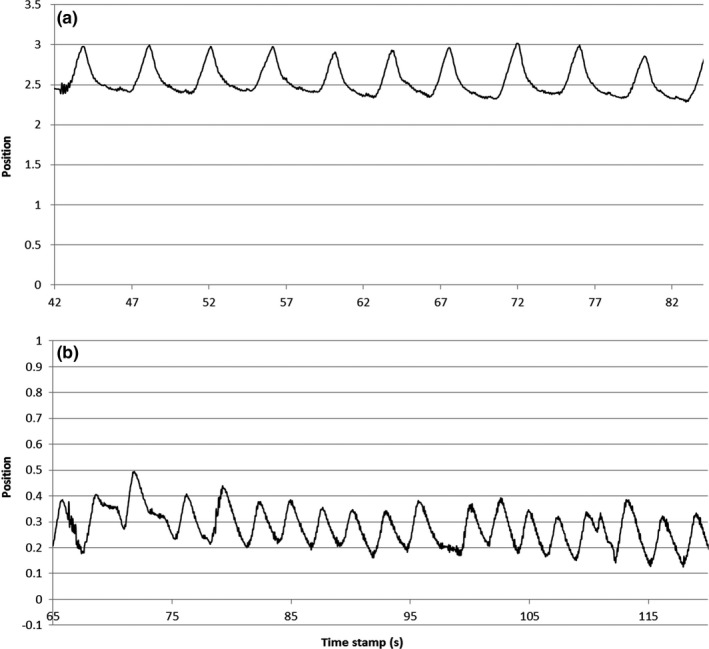
Sample respiratory traces from a patient who was administered general anesthesia (a) and one without (b).

For both inhale‐exhale displacement and free‐breathing margins, patients were analyzed in subgroups — general anesthesia vs. no anesthesia, age < 3 vs. age ≥ 3, and abdominal surgery vs. no surgery. Patients treated with anesthesia had very regular breathing in both frequency and amplitude while those who did not were not as uniformly consistent (Figure 4). This did not translate to a difference in magnitude of organ motion between the two groups. No significant differences between patient based on age or abdominal surgery were observed, as these sub‐analyses had too few patients to detect a statistically significant difference.

## 4 Discussion

Organs at risk contoured on 4DCT scans from 15 pediatric high‐risk neuroblastoma patients were evaluated to better understand pediatric organ motion. In analyzing the motion, the goal was to determine whether information provided by 4DCT could be reliably incorporated into treatment plans and whether standard margins for the pediatric population could be determined in order to reduce unnecessary exposure of normal organs. In some situations, tumor motion may be estimated through extrapolation of these data, for patients with intact tumors that are situated proximal to the normal organs at the time of radiotherapy.

Our analysis of organ motion demonstrated that, overall, inhale and exhale accurately characterized the motion extremes, and thus represented organ motion along the three axes (Figure 3),^8^, ^9^ consistent with previous literature addressing the adult population.

As the values in (Table 1) indicate, motion of the liver and spleen is closely correlated with that of the diaphragm (Figure 3), and larger than that of the kidneys. The correlation is strongest in the superior direction, and, interestingly, the superior displacement exceeded inferior displacement in the spleen, liver, and right diaphragm (the organs with the highest magnitude of superior displacement), which is consistent with liver findings by Hallman et al. This is also reflected in the muted movement of the centroid in the Y (S/I) direction. A mismatch of a margin with overall organ motion could indicate a volume deformation, specifically a compression. This was outside the scope of this paper, but could be investigated as a future extension using a 3D model to better understand organ deformation. This is an important area of future work because there are some organs that may have large respiration‐induced deformations, including the lungs. Despite excluding deformations, however, this work still has utility. Motion was characterized based on the edges of the organ, which provides a measure of the extent the organ would or would not move out of the radiation field. This has practical value regardless of the deformation, although deformation information would provide further information to characterize organ motion.

The kidneys exhibited the least motion, consistent with their retroperitoneal nature. They also showed far weaker correlations with diaphragm motion than did the liver and spleen. This has been observed previously.^12^ Excluding the diaphragms, organs within the abdomen moved no more than 4.5 mm while organs within the thorax moved no more than 7.4 mm (Table 1). Ultimately, the general pattern of abdominal motion was similar across the study population, with organs moving superiorly with exhale and inferiorly with exhale. The motion magnitudes, however, were difficult to predict.

Importantly, 4DCT provides respiration‐synchronized data, and caution should be applied in attributing organ motion fully to respiration. This is most notable in cardiac motion, where presumably motion arises from both respiration and myocardial contraction.

After assessing movement in the 4DCT scans, free‐breathing scans were analyzed with a goal of determining whether a reliable margin could be added to each organ in all three axes to fully encompass the free‐breathing organ. Such a “margin recipe” would prove valuable for institutions without 4D capability. Unfortunately, a reliable margin that held up to statistical rigor could not be determined. Even without error propagation, the liver and lung showed small margins of clinical interest, and organs on free‐breathing scans were often larger due to motion artifacts, blurring, and different scan acquisition time, contributing to difficulties with accurate contouring. The potential for a free‐breathing scan to be collected at any breathing phase also introduces a bias potential. These qualities support both the superiority of 4DCT in determining organ motion and the need for 4D treatment‐planning, particularly when treatment modalities that are sensitive to motion, such as proton therapy, are utilized. As there is a movement to image judiciously and with less radiation dose in the pediatric population, the need for more accurate imaging to reduce therapeutic side effects must be weighed against the increased imaging dose to the patient. While we have shown that the motion in pediatric patients is smaller than in adults, the use of 4DCT may result in smaller therapeutic margins and decreased radiation to OAR. Thus, as a result of this study, we recommend motion management for the pediatric population with neuroblastoma with consideration of 4DCT as the modality. In the future, if the set of data for pediatric patients were large enough for each organ, population data derived from 4DCT could be used. Until then, 4DCT data from each patient is what we recommend as a result of this study. If a free‐breathing scan is ultimately used for contouring, it is important to note that even the most extreme values in this study are still generally smaller than the displacements in adults.

The concept of error propagation was also used in this work. Most previous work evaluates only mean and SD, with the idea that a variance larger than the mean implies a lack of consistent motion trend. The inherent finite resolution of any measuring tool is not addressed by such an approach, which can be addressed by calculating the strength of motion after propagating error. Statistically, accounting for uncertainties in the contouring software allows for a more robust conclusion regarding the displacement between two imperfect positions. Organ motion that was significant despite error propagation is less likely to be due to inaccuracies in measurement or imprecision; however, may be more difficult to compare to previous literature. For this reason, we have supplied analysis both of motion significant in spite of error propagation and that significant without error propagation.

Organ motion has been studied extensively in adults,^5^, ^8^, ^13^ and published results vary widely. In large reviews, liver motion in individual studies has ranged from 8 mm to 25 mm on average, as compared to our study which found the maximum motion in any dimension of the liver to be 4.5 mm on average in the pediatric population.^13^ Kidney motion has ranged in the adult population from as little as 2 mm to 40 mm, with averages of approximately 10–20 mm.^13^, ^14^ Kidney motion in our pediatric population ranged from fractions of a mm to 2 mm, but overall smaller than any average found in the adult population, and often smaller than the minimal kidney motion in adult patient series. The same holds true for diaphragmatic motion, where adult motion values are on the order of 10 mm on average. Diaphragmatic motion observed in our pediatric population showed a maximum of approximately 8 mm with an average of 4–5 mm.^13^ However, because the method of contouring the diaphragm varies with each study, we are less able to directly compare the motion findings. Finally, splenic motion in the adult population has been observed to be similar to that of other large abdominal organs such as the liver, with averages around 2 cm.^13^ Again in our population, the motion was smaller for the spleen.

In contrast to the adult population, little work has been done on pediatric organ motion. Our study is the first to demonstrate that organ displacement due to respiration in children is much smaller than that observed in adults. This is a meaningful, if not altogether unexpected, result, implying smaller margins may be used in this population. Our findings were consistent with observations from the adult organ motion literature that maximum displacement is along the superior/inferior axis.^8^, ^13^, ^14^, ^15^, ^16^, ^17^ The findings in this paper are broadly applicable to pediatric cancers with tumors in the low thoracic or abdominal cavities. In our current work, neither age nor the presence of anesthesia, factors unique to the pediatric population, were significant factors in organ motion. Although our analysis did not directly examine tumor motion (because radiotherapy was delivered following tumor resection), extrapolation from data regarding normal organ motion suggests that internal target volumes (ITV), which are designed to take into account respiratory and other internal motion of the tumor and surrounding organs, may require modification within the pediatric population. Future study regarding pediatric patients with intact tumors would be of great interest and could influence practice to this end.

Our results raise several further questions. The analysis of organ motion via positional extremes, as shown in this work, is integral to margin determination.^10^, ^18^ A MidP strategy, which uses the mean position of an organ throughout the respiratory cycle as the planning volume, margin has been studied in lung volumes and has been found to result in smaller volumes and less therapeutic radiation dose.^19^ This finding is encouraging, especially given the significantly higher doses of radiation that accompany the use of 4DCT imaging.^20^ There are alternatives to 4DCT, including 4D‐MRI which, although not widely used or studied in relation to radiotherapy, that may minimize radiation exposure in the future.^21^ As discussed earlier, while our work shows that organs tend to move primarily in the superior/inferior direction, determining a 3D model would also be of value, as would investigation of motion of other organs within the pediatric population. The field of organ motion, especially in pediatric patients, is ripe with opportunity for future study.

## 5 Conclusions

While organ motion has been studied extensively in adults, there are far fewer investigations in the pediatric population. In this study, motion observed in children was of a smaller magnitude than that reported in adults. Pediatric organ motion also paralleled other general observations in adults, particularly that organ motion is greatest in the superior/inferior direction. We were unable to generate a corrective margin for free‐breathing scans that encompassed organs in all breathing phases, and thus 4DCT is an effective tool to accurately characterize organ motion in the pediatric population. This can result in result in more accurate tumor margins, ensuring target coverage while minimizing dose to organs at risk.

## Conflict of Interest

The authors have no conflict of interest.

## References

[1] Kry SF , Salehpour M , Followill DS , et al. The calculated risk of fatal secondary malignancies from intensity‐modulated radiation therapy. Int J Radiat Oncol Biol Phys. 2005;62:1195–1203. 15990025 10.1016/j.ijrobp.2005.03.053

[2] Hattangadi JA , Rombi B , Yock TI , et al. Proton radiotherapy for high‐risk pediatric neuroblastoma: early outcomes and dose comparison. Int J Radiat Oncol Biol Phys. 2012;83:1015–1022. 22138463 10.1016/j.ijrobp.2011.08.035

[3] Hill‐Kayser C , Tochner Z , Both S , et al. Proton versus photon radiation therapy for patients with high‐risk neuroblastoma: the need for a customized approach. Pediatr Blood Cancer. 2013;60:1606–1611. 23737005 10.1002/pbc.24606

[4] Pai Panandiker AS , Beltran C , Billups CA , McGregor LM , Furman WL , Davidoff AM . Intensity modulated radiation therapy provides excellent local control in high‐risk abdominal neuroblastoma. Pediatr Blood Cancer. 2013;60:761–765. 23024112 10.1002/pbc.24350

[5] Langen KM , Jones DTL . Organ motion and its management. Int J Radiat Oncol Biol Phys. 2001;50:265–278. 11316572 10.1016/s0360-3016(01)01453-5

[6] De Bruin PF , Ueki J , Bush A , Khan Y , Watson A , Pride NB . Diaphragm thickness and inspiratory strength in patients with duchenne muscular dystrophy. Thorax. 1997;52:472–475. 9176541 10.1136/thx.52.5.472 PMC1758554

[7] Rahman NNAA , Sridharan R , Singh DKA , Lee R , Sharif NBM . (2012). Quantification of diaphragm muscle thickness using ultrasound imaging: a preliminary study Proceedings of the 6th International Conference on Rehabilitation Engineering\&Assistive Technology, Tampines, Singapore pp. 41:1–41:3.

[8] Tai A , Liang Z , Erickson B , Li XA . Management of respiration‐induced motion with 4‐dimensional computed tomography (4DCT) for pancreas irradiation. Int J Radiat Oncol Biol Phys. 2013;86:908–913. 23688811 10.1016/j.ijrobp.2013.04.012

[9] Hallman JL , Mori S , Sharp GC , Lu HM , Hong TS , Chen GT . A four‐dimensional computed tomography analysis of multiorgan abdominal motion. Int J Radiat Oncol Biol Phys. 2012;83:435–441. 22197238 10.1016/j.ijrobp.2011.06.1970

[10] Huang L , Park K , Boike T , et al. A study on the dosimetric accuracy of treatment planning for stereotactic body radiation therapy of lung cancer using average and maximum intensity projection images. Radiother Oncol. 2010;96:48–54. 20430460 10.1016/j.radonc.2010.04.003

[11] Clements N , Kron T , Franich R , et al. The effect of irregular breathing patterns on internal target volumes in four‐dimensional CT and cone‐beam CT images in the context of stereotactic lung radiotherapy. Med Phys. 2013;40:021904. 23387752 10.1118/1.4773310

[12] Sharma S , Naik M , Hua C , Krasin MJ , Merchant TE , Pai Panandiker AS . Renal motion in pediatric patients measured with respiratory correlated 4D‐CT. Int J Radiat Oncol Biol Phys. 2009;75:S513–S514.

[13] Yamashita H , Yamashita M , Futaguchi M , et al. Individually wide range of renal motion evaluated by four‐dimensional computed tomography. Springerplus. 2014;3:131‐1801‐3‐131. eCollection 2014. 24711985 10.1186/2193-1801-3-131 PMC3977021

[14] Langen K , Jones D . Organ motion and its management. Int J Radiat Oncol Biol Phys. 2001;50:265–278. 11316572 10.1016/s0360-3016(01)01453-5

[15] Kubas A , Mornex F , Merle P , d'Hombres A , Lorchel F , Chapet O . L'irradiation des carcinomes hepatocellulaires: impact de la respiration sur les mouvements et variations de volume de la tumeur, du foie et des organes intra‐abdominaux. Cancer/radiotherapie. 2008;12:768–774. 10.1016/j.canrad.2008.04.011 18639479

[16] Brandner ED , Wu A , Chen H , et al. Abdominal organ motion measured using 4D CT. Int J Radiat Oncol Biol Phys. 2006;65:554–560. 16690437 10.1016/j.ijrobp.2005.12.042

[17] Gawthrop JB , Gill S . The use of respiratory‐correlated four‐dimensional CT where kidney motion has the potential to impact upon the radiotherapy planning process. J Med Imaging Radiat Oncol. 2012;56:689–695. 23210590 10.1111/j.1754-9485.2012.02458.x

[18] Muirhead R , McNee SG , Featherstone C , Moore K , Muscat S . Use of maximum intensity projections (MIPs) for target outlining in 4DCT radiotherapy planning. J Thorac Oncol. 2008;3:1433–1438. 19057269 10.1097/JTO.0b013e31818e5db7

[19] Wanet M , Sterpin E , Janssens G , Delor A , Lee JA , Geets X . Validation of the mid‐position strategy for lung tumors in helical TomoTherapy. Radiother Oncol. 2014;110:529–537. 24424385 10.1016/j.radonc.2013.10.025

[20] Matsuzaki Y , Fujii K , Kumagai M , Tsuruoka I , Mori S . Effective and organ doses using helical 4DCT for thoracic and abdominal therapies. J Radiat Res. 2013;54:962–970. 23603303 10.1093/jrr/rrt024 PMC3766296

[21] Shimizu S , Shirato H , Aoyama H , et al. High‐speed magnetic resonance imaging for four‐dimensional treatment planning of conformal radiotherapy of moving body tumors1. Int J Radiat Oncol Biol Phys. 2000;48(2):471–474. 10974464 10.1016/s0360-3016(00)00624-6

